# Compassionate use programs in Italy: ethical guidelines

**DOI:** 10.1186/s12910-018-0263-8

**Published:** 2018-03-09

**Authors:** Ludovica De Panfilis, Roberto Satolli, Massimo Costantini

**Affiliations:** 1 Direzione Scientifica, AUSL di Reggio Emilia-IRCCS, Viale Umberto I 50, Reggio Emilia, Italy; 2 Comitato Etico dell’Area Vasta Emilia Nord, Viale Umberto I 50, Reggio Emilia, Italy

**Keywords:** Compassionate use programs, Ethics committee, Cancer, Quality of life, Right to hope, Simultaneous palliative care

## Abstract

**Background:**

This article proposes a retrospective analysis of a compassionate use (CU), using a case study of request for Avelumab for a patient suffering from Merkel Cell Carcinoma. The study is the result of a discussion within a Provincial Ethics Committee (EC) following the finding of a high number of requests for CU program. The primary objective of the study is to illustrate the specific ethical and clinical profiles that emerge from the compassionate use program (CUP) issue. The secondary goals are: a) to promote a moral reflection among physicians who require approval for the CUP and b) provide the basis for recommendations on how to request CUP.

**Main body:**

The instruments for carrying out the analysis of the case study and the discussion are as follows:

Analysis of the audio-recording of the EC meeting regarding the selected Case study.

In-depth discussion of topics that emerged during the meeting by means of administration of 5 semi-structured interviews with 2 doctors involved in the case (proposing physician and palliative physician) and with 3 components of the EC who played a major role in the EC internal discussion.

**Conclusions:**

In an exploration of emerging clinical and ethical issues, four primary themes arise: 1. efficacy, safety of the treatment and patient’s quality of life; 2. clear, realistic, adequate communication; 3. right to hope; 4. simultaneous Palliative Care approach. The results of ethical analysis carried out concern two areas: 1) ethical profiles relating to the use of CUP; 2) the role of the EC concerning the compassionate use of drugs and the need to provide recommendations on how to request CUP.

With the aim of implementing these conclusions, the provincial EC of Reggio Emilia chose to steer the request for drugs for compassionate use through recommendations for good clinical and ethical practice based on the following assumptions: 1) the “simultaneous care” approach must be preferred. Secondly, 2) the EC’s assessment must be part of the decision-making process that the care team conducts before proposing compassionate use to the patient.

## Background

Compassionate use (CU), also referred to as expanded access, is the therapeutic use of investigational drugs outside of clinical trials. According to the definition of the European medicines Agency (EMA) “compassionate use is a treatment option that allows the use of an unauthorized medicinal product that is under development” [1]. Eighteen out of the 28 European states (64%) have well-defined national regulations and procedures for the compassionate use of drugs [2].

In Italy, the use of drugs for nominal therapeutic purposes is regulated by the Decree of 8 May 2003 “Therapeutic use of medicinal products subjected to clinical trials” [3], which describes the methods for requesting the drug and the conditions in which authorization may be issued. According to this regulation, a drug can be requested for use outside clinical trial “when there is no valid therapeutic alternative to the treatment of serious illnesses, or rare diseases, or disease conditions that put the patient’s life at risk” [3]. The conditions under which authorization for use of the medicinal product may be issued are: 1) a phase 3 trial aimed at assessing the effectiveness of the medicinal product is ongoing or completed; 2) in special cases when illness puts the patient’s life at risk, a completed phase 2 trial can be considered enough; 3) the data available concerning the trials must be sufficient to form a favorable opinion regarding the efficacy and the tolerability of the drug [3]. Lastly, the request must necessarily be subjected to the opinion of the Ethics Committee (EC) in the context in which it originates and for which authorization must be obtained. As reported in a recent paper [4] only a few countries, including USA, Spain and Italy, require EC approval for compassionate use. According to the authors, there are two key arguments for the mandatory ethical review of compassionate use: 1) Compassionate use may involve significant research aspects; 2) compassionate treatment is based on drugs with unproven safety and efficacy, which require an evaluation of the risk-benefit ratio [4].

The role of the EC in the process for request and authorization (or no authorization) for CU is crucial, especially in the presence of ethical dilemmas that emerge frequently, due to, for example, disagreement of opinions within the EC regarding the approval of the drug. The absence of an evidence-based reference frame-work involves decision-making procedures that are not always univocal and, often, linked to the dynamics of the individual case.

The clinical and ethical significance of the issue of unauthorized treatments also finds its basis in the Declaration of Helsinki - ART.37: In the treatment of an individual patient, where proven interventions do not exist or other known interventions have been ineffective, the physician, after seeking expert advice, with informed consent from the patient or a legally authorized representative, may use an unproven intervention if in the physician’s judgement it offers hope of saving life, re-establishing health or alleviating suffering. This intervention should subsequently be made the object of research, designed to evaluate its safety and efficacy. In all cases, new information must be recorded and, where appropriate, made publicly available [5]. As clearly described in Art. 37 of the Declaration, the prima facie ethical issues concern the patient’s consent, decision-making capacity, right to hope, and quality of life (QoL). Generally, we can affirm that the ethical issues regarding the CU can be traced back to three classical principles: justice, charity and autonomy [6], although the individuality of the patient and the singularity of the disease also play an equally decisive role.

In this study, we present a retrospective analysis and an ethical evaluation of the decision-making process of the Provincial EC of the AUSL – IRCCS of Reggio Emilia, Italy, concerning a specific CU request for Avelumab for a patient suffering from Merkel Cell Carcinoma.

The project was the result of a discussion within the EC following the observation of a high number of Requests for CU. In the period 2015–2016 the EC received 86 requests for Nominal Therapeutic Use, of which 33 were in 2015 and 52 in 2016. The reference population for the Reggio Emilia Province is 533,827 persons in the year 2016. All these requests received EC approval, in spite of the high degree of problems of some of these. Moreover, the large number of requests is shared with other Italian ECs: the EC of the Bologna University Hospital recently conducted a retrospective analysis of requests for compassionate use in the period 2010–2015. In 5 years, the EC received 610 requests for a reference population of some. 873,471 persons [7].

Given the ethical and deontological implications of the CU issue, we chose an example of a Case study to which a general ethical analysis scheme was applied **(**Table 1**)** [8–10]. An ethical analysis is applied to ethical dilemmas which arise when there is a conflict of values as to which is the right decision to be made in a clinical context [10]. More specifically, “a dilemma is a decisional conflict occurring within a single agent whenever one must decide between two or more mutually exclusive courses of action, so that selecting an option necessarily results in discharging the other. The peculiar feature characterizing ethical dilemmas is that the reasons that the agent provides in favor of one of the two alternatives are specifically moral reasons, that is, reasons concerning moral principles and values” [9, 10].

**Table 1:** Ethical analysis of the case

1. Collecting data and defining the terms used
• medical aspects (current standard, diagnostic and treatment options, benefits and risks), psychological, relational issues
2. Definition of the ethical principles at stake and of the various responsibilities
• actors involved in addition to the patient, legal figures, degree of autonomy of the patient and of the persons involved, informed consents and recognition of national and international legal standards
3. Clarification of conflict of interests and identification of the ethical problems
• ethical problems • conflict between principles, team conflicts, conflicts between patient/family and care personnel
4. Evaluation of possible options
• Analyses of possible courses of action
5. Justification of choice
• Course of action and ethical principle • Preferences for one course of action over another • Resolution of conflict between values • Principles and facts

The instruments for carrying out the analysis of the case study and the discussion are as follows:

- analysis of the audio-recording of the EC meeting regarding the selected Case study;
- in-depth discussion of the topics emerged during the meeting by means of administration of 5 semi-structured interviews to 2 involved physicians (the oncologist who requested the drug and the Palliative Care (PC) specialist) and to 3 components of the EC. We chose to interview the PC specialist after the analysis of the audio-recording of the EC meeting, as explained below in the text. The components of the EC who played a major role in the ethical discussion were selected for the interview. Written informed consent was obtained from the participants for collecting and analysing data before the interview.
- The EC meeting and Interviews were audio-recorded and transcribed verbatim. The authors analysed transcriptions, mapping the following analytical stages according to the standard methodology for qualitative analyses [11]:
- A researcher (LDP) transcribed the interviews verbatim and shared the transcripts with colleagues so that they could become familiar with them. They wrote comments and initial thoughts in a memo;
- Provisional themes were identified. Subsequently, the researchers independently reviewed themes and allocated portions of the text to those themes;
- All the authors re-defined themes and re-named them to achieve consistency in the definition of the proposed themes.

The main topics that emerged from the interviews are listed in Table 2.

**Table 2:** Main issues emerging from the interviews

Subjects interviewed	Topic
Three components of the EC	Increasing number of requests
	Absence of exceptions to the procedure
	Absence of good clinical practice
	Safety tests and guarantee of efficacy often lacking
	Not adhering to MD 2003
	Specific case:
	Multiple Bias of the study of the drug requested
	Problem of patient information
	Importance of an informed decision and the activation of PC
	Simultaneous PC
	Right to hope
	Quality of life
Proposing physician	Patient already self-informed
	Patient in good general conditions
	excellent evaluation of the PC intervention at the EC’s request
Palliative Care Specialist	Simultaneous PC
	Patient awareness, informed decision
	Guarantee quality of life
	Specific case:
	Risk of misunderstanding of PC
	No continuity of patient management before and after the interview regarding compassionate requesting

The study was approved by the Arcispedale Santa Maria Nuova Provincial Ethics Committee of Reggio Emilia (Protocol n. 2016/0028509, November, 30th, 2016).

The primary objective of the study was to provide a clinical/ethical decision-making analysis that illustrates the specific ethical and clinical profiles emerging from the case study. The secondary goals are: a) to promote a moral reflection among physicians who require approval for the CU and b) provide the basis for recommendations on how to request CU.

## Case study

The EC examined the request by an Oncologist for Therapeutic Use of the drug Avelumab for a patient (age range 65–69) suffering from Merkel Cell Carcinoma. The patient was in good conditions at the time of the request, but with progressive disease for which there are no therapeutic alternatives.

The Hospital Pharmacy had previously assessed the request as not conforming to the requirements envisaged by Italian Ministerial Decree (MD), 2003. The EC therefore made a specific request to the Italian medicine Agency which confirmed the opinion of the Hospital Pharmacy with respect to the drug administration.

Despite these answers, the Director of the Oncology Unit requested that the case be presented and discussed with the EC.

At this point, the EC asked for an official meeting with the proposing oncologist, to evaluate not only the regulatory aspects but also several clinical and ethical perplexities regarding drug approval.

During the meeting, it came to light that there was no documented efficacy and guarantee of safety for this drug, as we describe in detail in the next paragraph. Despite clinical evidence, contextual issues also emerged that the EC decided to examine in detail. In particular, the proposing oncologist reported that the patient was already aware of the possibility of the drug as well as of the request for approval of the EC. During the EC meeting and the interview, emerged that the oncologist explained to the patient the possibility of an experimental treatment presented as “the only option.” He also said to the patient that the only way to have access to the treatment is the EC approval. At the time of the meeting, the patient was awaiting a reply and this condition inevitably changed the opinion parameters. The discussion was therefore focused on certain central questions which will be discussed in detail in the following paragraphs: the ethical principles at stake and their balance; the search for a solution that respects not only the clinical, but also the moral interests involved; the need for definition of good practices with respect to the demand for drugs for compassionate use.

In particular, the ethical dilemma facing the EC can be summarized as follows: is it right to authorize the drug with the only motivation not to deny hope for the patient who is waiting for an approval from the EC or is it more correct to protect the patient from irresponsible administration of a drug the efficacy of which has not been proved? (while at the same time fueling imprudent hope?).

The following ethical analysis aims to clarify the deliberative process of the EC and motivate the decision that was finally made.

## Discussion and ethical analysis

The following analysis describes the deliberation process of the EC of a research hospital as regards a controversial request for compassionate use of a drug. Given the problems that emerged, the decision of the EC was particularly disputed. The clinical, ethical and decisional profiles of the discussion were as follows: 1. Treatment efficacy and safety and patient’s QoL; 2. clear, realistic, adequate communication; 3. right to hope; 4. the role of Palliative care (PC) specialist. The steps of the ethical analysis used are as follows: analysis of the ethical profiles; indication of the possible courses of action; justification of the choice.

### Drug efficacy, safety and patient’s quality of life

The in-depth analysis of the semi-structured interview of one of the components of the EC revealed that the request for compassionate use of Avelumab in a patient suffering from metastatic Merkel Cell Carcinoma was based on a Phase II (JAVELIN Merkel 200), single-arm, open-label single study conducted on 88 patients and sponsored by the drug manufacturer. At the time of the interview, the results of the study had not been published in peer-reviewed scientific journals but were presented only in the form of abstract at a recent congress of the American Society of clinical Oncology (ASCO). The extent of the primary outcome was identified as a surrogate endpoint and subject to bias (the overall response rate ORR) and in this case statistically and clinically not relevant; the Progression Free Survival (PFS) with Avelumab is 2.7 months and is consistent with the historic data reported for chemotherapy (61 days). The only apparent advantage was the 6-month durable response rate (29.1% vs. 6.7%), an evidence that was based on a surrogate outcome not appropriate for evaluating the efficacy of immunotherapy.

There are numerous ethical questions: without prejudice to the instructions of the MD to be followed, is it right to deny a treatment in the name of safety, when the patient’s only certainty is that of dying in a short time? What role does the concept of QoL have in this clinical and decision-making context? When are compassionate treatments likely to turn into a form of aggressive or futile treatments? Who decides the adequacy of a treatment and on the basis of what assessments?

The personal QoL and the concept of futile treatment refer to a subjective sphere of values of the individual which primarily concerns, in the first place, the principle of decision-making autonomy. The health-related quality of life (HRQOL) [12] is a multidimensional concept that includes a set of physical and physiological, functional, existential and social conditions that have an influence on the health of an individual [13]. The futile treatment is closely connected with the concept of QoL. The Italian National Committee of Bioethics (CNB) in 2008 defined futile treatmen as: “a disproportion between the efficacy and the severity of the treatments practiced and the benefits that can be obtained under concrete clinical circumstances (in this regard, “futile treatments”), it being understood that every treatment must be evaluated balancing the potential positive (benefits) or negative inputs” [14]. The importance of the subjective component of these issues and the need to consider a series of factors is obvious, like the relational context in which the patient’s clinical history is inserted: correct information provided to the patient as well as the acknowledged focus of his/her wish are elements indispensable for the concrete implementation of the principle of autonomy, because these allow the sick person to make a conscious choice. As Christman suggested, making choices is one of the four-way to intend autonomy: autonomy is a fundamental right, it is a basic concept of liberal political theory, it is the way to realize integrity and dignity and, lastly, it is the capacity of the competent moral agent [15]. The common theme is the ability to make choices as “self-government,” as expression of human freedom [15]. The definition of autonomy proposed by Beauchamp and Childress [16] states: “Autonomy is the personal rule of the free-self (...). The principle of autonomy requires respect for the choices made by individuals whose decisions are taken freely” [16]. The deriving ethical choice concerns the method of composition between the lack of proven efficacy of a drug and the efficacy which, on the other hand, the patient claims to experience.

### Clear, realistic, and adequate communication

“Monitoring the use of words is an ethical imperative” [17], especially in case of an incurable disease: the information given to the patient must be as appropriate, clear, realistic, and individualized as possible. As stated by the Italian Medical Code of Ethics, “The communication time is the treatment time”, [18] and numerous studies show that good clear and realistic communication influences the well-being of patients, doctors and all healthcare operators, and can contribute to better control of the symptoms, increasing adherence to treatments [19–22].

Adequacy of the communication to the patient must also be time-related: together with clarity, honesty and individualization, gradualness is also an element necessary for the decision-making process of the sick person regarding the treatment choices that concern him/her directly. In the case study described here, the subject of the debate was the method of communication and the choices made by the patient. How can the patient’s awareness and his/her understanding of the options available be verified? Moreover, if it is true that “patients should have a right to mitigate extreme suffering and to enhance self-preservation and that patients are presumed to be capable of making well-informed treatment decisions in consultation with their physicians”, it is equally true that “data on experimental drugs are very limited and they do not have the training or experience to evaluate the combined pharmacologic, clinical, and statistical information on experimental therapies that is available to them” [23].

The choice of CUP to be proposed must always be carefully evaluated by the treatment team and, subsequently, must be communicated correctly and suitably to the patient together with the rest of the treatment options available, considered within a shared treatment plan and as the expression of correct shared decision making.

### Right to hope

The question of a correct communication outlined above is closely connected to the timing of the information and its adequacy as regards the request for CUP.

Informing the patient of this treatment option before its actual approval by the EC is a widespread practice. This attitude changes the scenario of the patient’s choice radically: first, for the sick person who is given a hope that cannot yet be confirmed and, second, for the EC, which must make a decision that is no longer free of constraints. In the case in question, the patient’s right to hope was one of the main topics of discussion, because, as claimed by one of the EC members interviewed, refusal – albeit motivated – “is inevitably seen by the patient as denial of the last hope”.

The issue of “right to hope” is controversial: according to the CNB there is no right to hope, but only the “feeling of hope”, which must be taken care of by controlling “how” and “by whom”the patient is assisted as well as by ensuring clear and truthful information. As claimed by relational ethics, good treatment demands truth, because “the real word, even when it produces pain, has positive consequences as it triggers a process of critical self-understanding” [17].

The right to hope is undoubtedly linked to the quality of life. In the semi-structured interview, the EC chairman states that “we cannot hope not to die, but we can hope to live well right up to the end. Therefore, the double approach, palliative and curative, is necessary. The palliative approach must not be that which evokes the end of hope: the fundamental error lies in not activating Palliative Treatments first”.

### Are simultaneous palliative care the better solution?

In the light of what has been described above, the EC suggested to suspend the decision until activation of the intra-hospital PC team, in order to better assess the patient’s awareness of the disease, his/her knowledge of the possible alternatives, his/her wishes and information regarding the experimental treatment proposed. It was an “urgent” activation of the PC team that highlighted some of the critical issues reported by PC Specialists during the in-depth interview: if, on the one hand, involvement of the PC in this phase was considered as very positive by the oncologist interviewed, according to the PC Specialist, the solution does not seem to meet the requirements of a real approach of Simultaneous PC or early PC. According to the doctor interviewed, the activation of PC at this stage of the care path is likely to create misunderstandings regarding the role and the mission of the PC. The PC Specialist reported the absence of continuity of patient management following the interview. Fostering an early palliative approach requires follow-up to diagnosis, or, in any case, certainly before all the treatment options available run out: the randomized study by Temel in 2010 not only showed that the average survival is higher in the group of patients who receive palliative treatment immediately after diagnosis, but that Early PC also have a positive effect on the QoL and depressive symptoms [24]. While admitting that the treatments cannot or must not always be started simultaneously immediately after diagnosis of the tumor, numerous studies point out that the PC must not be the last resort treatment, after which the patient has run out of all the treatment options available. For a simultaneous palliative approach to be preferred, the PC must be activated early and not as a tool for EC verification on the process for request for compassionate use of the drug.

After counselling by the PC Specialist who confirmed the patient’s awareness and information and the willingness to proceed with a further therapeutic attempt, the request was approved and the drug was administered regularly. At the time we are writing, the patient can benefit from the use of the drug.

As reported by one of the components of the EC during the in-depth semi-structured interview, “the request was inadmissible under law, but after consultation with the PC Specialist who ensured that the patient was already aware of and had been informed of this possibility, if we had refused the request, we would have caused certain, obvious and real damage to the patient, as against clinical harm relative to the obvious less certain harmfulness”. In the first case, it could have been considered as a mainly emotional and moral damage; in the second hypothesis, the damage was potentially clinical.

The EC deliberation was found to be focused on the process of making the decision regarding the patient through dialogue with the PC Specialist, respecting the principle of relational autonomy. It seeks to consider the influence on individual opportunities that interdependence and subjective vulnerability have. In addition, relational autonomy reveals several ways in which autonomy is specifically conditioned by social structures [25]. Dignity, respect, empathy and care are key concepts for the definition of relational autonomy [26]. Moreover, the EC deliberation also focused on the expression of a truly informed consent and guaranteeing self-determination in the choices of treatment.

## Conclusions

The results of the ethical analysis carried out concern two areas: 1) ethical profiles related to the use of CUP and, 2) the role of the EC regarding the compassionate use of drugs and the need to provide recommendations on how to request CUP.

Numerous ethical issues have emerged that deserve to be studied in detail: the balance between treatments efficacy/safety and QoL, the importance of a clear, realistic, adequate communication, the right to hope and simultaneous PC. The case study presented also contributes to the discussion of the decision-making role of institutions in the final stages of the life of individuals, the main topic of the bioethical and legal debate [27, 28]. The EC must promote the awareness of the decision-making patient, and secondly, provide the doctor with ethical advice regarding good clinical practice.

With the aim of implementing these conclusions, the EC of Reggio Emilia chose to steer the request for drugs for nominal therapeutic use through recommendations for good clinical and ethical practice based on the following assumptions: 1) the “simultaneous care” approach must be preferred. In fact, it helps to provide a more complete and realistic picture of the disease, the prognosis and the possibilities of curing and/or the quality of remaining life being higher than that proposed only by the oncologist’s point of view. Secondly, 2) the EC’s assessment must be part of the decision-making process that the care team conducts before proposing compassionate use to the patient. Without the preliminary ethical evaluations by the EC, the proposal may be premature or imprudent, since the possibility of experimental therapy has not yet been verified.

In order to ensure that physicians follow these recommendations, the EC requests the proposing physicians to attach two documents to the request for nominal compassionate use: 1) the PC consultancy which certifies management of the patient and helps spread the culture of the simultaneous palliative approach in hospital, in order to avoid urgent activation as in the case study described or non-activation; 2) a declaration in which the physician states that the patient has not yet been informed of the possibility of yet another therapeutic possibility.

The purpose of these recommendations is two-fold: first, to allow the patient to receive a simultaneous approach of PC and to provide ethical and decision-making advice to the treatment team and second, to limit the number of applications and improper use of the CUP.

Future prospective studies can be aimed at exploring the qualitative and quantitative outcomes of simultaneous activation of PC.

## Abbreviations

PC: Palliative Care
CU: Compassionate Use
MD: Ministerial Decree
ASCO: American Society of clinical Oncology
PFS: Progression Free Survival
EMA: European medicines Agency
EC: Ethics Committee
PC: Palliative Care
